# Vitamin D receptor (BsmI) gene polymorphism and allele frequency among chronic kidney disease patients in south Indian population

**DOI:** 10.6026/97320630019380

**Published:** 2023-04-30

**Authors:** Ramya Badrachalam, Vadivel Mani, Ravi Kumar, Asmathulla Shafiulla

**Affiliations:** 1 Department of Biochemistry, Sri Manakula Vinayagar Medical College and Hospital, Puducherry - 605107, Tamil Nadu, India; 2 Department of Biochemistry, Konaseema Institute of Medical sciences and research foundation, Amalapuram, East Godavari Dt-533201, Andhra Pradesh, India; 3 Department of Biochemistry, All India Institute of Medical Sciences, Madurai- 625006, Tamilnadu, India

**Keywords:** Chronic kidney disease, BsmI gene polymorphism, allele frequency, genotypic frequency, glomerular filtration rate, single nucleotide polymorphism.

## Abstract

The vitamin D receptor (VDR) axis plays an important role in multiple physiological renal functions. BsmI gene is one among the VDR gene plays a vital role in maintaining this VDR axis and any polymorphism in VDR gene will cause dysfunction of renal tissues. The main objective of the study is to study the link between BsmI VDR gene polymorphism and Chronic Kidney Disease (CKD). This was a case-control study, which includes 100 cases and 100 controls. BsmI gene analysis was done by polymerase chain reaction - restriction fragment length polymorphism (PCR-RFLP). Among 100 CKD study participants, BB (wild-type) genotype of BsmI gene was present in 7 patients (7%), Bb (heterozygous) genotype was present in 23 patients (23%) and bb (mutant) genotype was present in 70 patients (70%). And among 100 controls, 92 subjects were found to have BB genotype and 8 subjects were found to have Bb genotype and none of subjects were found to have bb genotypes. CKD patients with Bb and bb genotypes were found to have significantly elevated serum urea, creatinine and decreased Glomerular Filtration Rate (GFR) when compared to the BB genotype of BsmI gene. 'b' allele of BsmI gene, Bb and bb genotypes of BsmI gene plays a greater role in Guanine/Adenine single nucleotide polymorphism of BsmI gene in CKD.

## Background:

Chronic Kidney Disease (CKD) is defined based on the two clinical criteria, first criteria includes glomerular filtration rate (GFR) < 60ml/min/1.73m2 for ≥ 3 months with or without evidence of kidney damage and the second criteria includes the evidence of kidney damage with or without decreased GFR for ≥ 3 months [1].CKD is a global threat for developing countries because of the expensive and lifelong therapy [2, 3]. With the global prevalence of CKD about 11 to 13% and worldwide incidence of 10%, recent study attempts to highlights that one in every 20 individuals is suffering from CKD and that too the prevalence is slightly higher in elderly age group, even there is a tendency for the younger people to be affected with CKD and its significant relationship with advancing age [4].Prevalence of CKD in India is about 17.2% with mean age of the population of about 45.22 ± 15.2 years. The stage wise prevalence of CKD, with stage 1 - 7%, stage 2 and 3 - 4.3% and stage 4 and 5 - 0.8% [5, 6]. The pathogenesis of CKD includes multiple genetic and environmental factors leading to end stage renal disease (ESRD) [7, 8]. Studies have shown that many single nucleotide polymorphisms (SNP) are associated with CKD [9, 10, 11, 12], the most common SNP was vitamin D receptor (VDR) gene polymorphism. Genetic studies have revealed that VDR genes are highly polymorphic and associated with development of CKD in addition to environmental factors. Vitamin D activation occur in kidney, so any alteration in the sequence of VDR gene can alter the normal renal function and predispose to development of CKD and its complications. VDR is known to mediate pleiotropic biological actions of 1,25- dihydroxy vitamin D3. VDR is a member of nuclear receptor family of transcription factor, it is also called as calcitriol receptor or nuclear receptor subfamily (group I and member 1) [13, 14, 15]. Human VDR gene located on chromosome 12q, consists of 11 exons along with introns and made up of 75kb (16). The four common SNP of VDR gene includes BsmI,ApaI, FokI and TaqI [17]. Bsm1 is located on intron 8 of chromosome 12q, consists of two different alleles (B,b) and its polymorphism is guanine/adenine (G/A) variation in intron 8 [18, 19]. Among these, four SNP, BsmI and ApaI have been identified as risk factors in the progression of CKD. The purpose of this study was to reveal the allelic and genotypic frequency among CKD patients in South Indian population.

##  Methods:

This was a case-control study done at Sri ManakulaVinayagar Medical College and Hospital (SMVMCH), from January 2018 to January 2019 after obtaining permission from Institutional Ethics committee (Code No: 45/2017). The study includes 100 CKD cases and 100 controls. A total of 100 CKD patients were recruited for the study from Nephrology Department and 100 controls were recruited from General Medicine out Patient Department (OPD) of SMVMCH, Puducherry. Informed written consent was obtained from all the study participants.

## Inclusion criteria for CKD patients:

CKD patients with age group 45 - 70 years were included in this study. 2. CKD patients with serum urea value > 40mg/dl 3. CKD patient's with serum creatinine > 1.2mg/dl 4. CKD patients with GFR <60ml/min/1.73m2 Where GFR was calculated using the modification of diet in rural disease formula (MDRD). 5. CKD patients undergoing hemodialysis were also included in this study. 6. CKD study participants were staged based on the KDIGO (Kidney Disease Improving Global Outcomes) CKD staging by using GFR [20, 21].

## Inclusion criteria for controls:

[1] Study participants with age group 45 - 70 years 2. Subjects with normal serum urea, creatinine and GFR were included in this study.

##  Exclusion criteria:

[1] Patients with acute renal failure

[2] Patients with renal cell carcinoma

[3] Sample size was calculated using the formula 4pq/d2 with previous CKD prevalence of 17.2% with 10% precision and 95% confidence level.

## Blood Sample collection:

After getting proper informed consent 2 ml of Ethylene Diamine Tetra-acetic Acid (EDTA) blood sample was collected from all the study participants for Deoxyribo Nucleic Acid (DNA) extraction [22]. CKD patients from Nephrology department and controls from General Medicine OPD were selected for the study using random sampling method.

## Genetic Analysis:

Genomic DNA was extracted by spin column method using QIAamp DNA mini kit according to the manufacturer's instructions [23]. DNA quantity was assessed by checking the absorbance of the extracted DNA using spectrophotometer.

The ratio of absorbance A260: A280 was 1.8 (this ratio value indicates that the extracted DNA was not contaminated by Ribonucleic acid and proteins).

DNA quantity was also assessed by spectrophotometer using the formula: DNA concentration (µg/ml) = (A260 reading - A320 reading)X dilution factor X 50µg/ml. Gradient Polymerase Chain Reaction (PCR) amplification was done to detect BsmI gene polymorphism using VDR (BsmI) Forward primer with 5'-CAACCAAGACTACAAGTACCGCGTCAGTGA- 3' (GC content = 50%, Tm = 68.1°C) and Reverse primer with 5'-AACCAGCGGGAAGAGAGGTCAAGGG- 3' (GC content = 60.9%, Tm = 66°C) (Manufacture name: Eurofins Genomics) to produce BsmI gene amplicon with 825bp length [24].

## Determination of BsmI genotype:

Thermo cycling consisted of denaturation at 95°C for 120 seconds, annealing at 65°C for 60 seconds and extension at 72°C for 180 seconds, for 30 cycles followed by final extension at 72°C for 5 minutes (25). PCR products were detected by 1% agarose gel electrophoresis with ethidium bromide stained and visualized by Ultra-Violet (UV) transilluminator. Then BsmI gene was subjected to restriction enzyme digestion for 1 hour at 37°C with BsmI restriction enzyme and the digested products represents the Restriction fragment length polymorphism (RFLP). This RFLP were detected by 3% agarose gel electrophoresis stained with ethidium bromide and visualized under UV transilluminator. The three different patterns of BsmI gene restriction fragments were obtained in agarose gel electrophoresis. BsmI gene without restriction sites will produce single fragment of 825bp (Homozygous wild - BB genotype), BsmI gene with restriction sites will produce either 2 fragments with 650bp and 175bp (Homozygous mutant - bb genotype) or produce 3 fragments with 825bp, 650bp and 175bp (Heterozygous - Bb genotype) respectively [26].

## Statistical Analysis:

Statistical analysis was done using SPSS software version 24.0. Allele and genotypic frequencies were done by direct gene counting method. Comparison of BsmI genotypes with the study variables among cases and controls were expressed as mean ± SD and were done using student t test. P values < 0.05 were considered significant.

## Results:

Table 1 shows the comparison of the study parameters between cases and controls. There were significant increase (p value < 0.001) in serum creatinine and urea in CKD cases than compared to the controls. And GFR was significantly decreased (p value < 0.001) in CKD cases when compared to the controls. Both cases and controls involved in this study were age and gender matched. And among 100 CKD study participants, 51 were males and 49 were females. CKD study participants were staged using KDIGO Staging criteria. Among these 100 CKD patients, 10 Patients were having stage-1, 13 patients were having stage-2, 8 patients were having stage-3a, 12 patients were having stage-3b, 50 patients were having stage-4 and 7 patients were having stage-5 of CKD respectively. Further, these 7 patients with stage-5 of CKD were on hemo dialysis.

**Figure 1:**
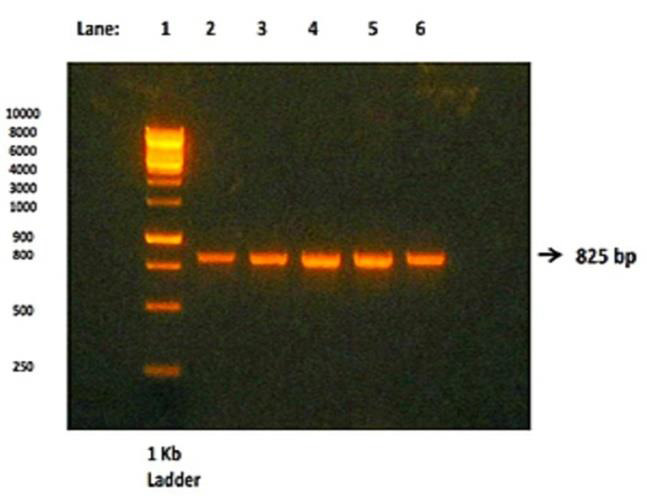
Agarose gel electrophoresis pattern of BsmI gene before restriction digestion.

**Figure 2:**
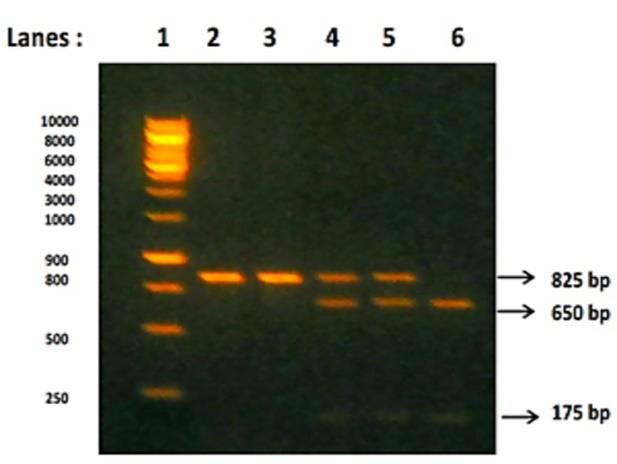
Agarose gel electrophoresis pattern of BsmI gene after restriction digestion in CKD patients

Table 2 and Figure 2, 3 shows the distribution of BsmI VDR gene polymorphism among the cases and controls. Out of the 100 CKD study participants, 7 (7%) patients were having BB genotype (Wild type without SNP), 23 (23%) patients were with Bb genotype (Heterozygous type with SNP) and 70 (70%) patients were with bb genotype (Homozygous mutant type with SNP). Among 100 CKD patients, 19% of patients were having 'B' allele and 81% 'b' allele of BsmI gene. Out of the 100 controls, 92 (92%) subjects were having BB genotype, 8 (8%) subjects were with Bb genotype and further none of the subjects in the control group were having bb genotype. Among 100 controls, 96% of subjects were having 'B' allele and 4% of subjects were having 'b' allele of BsmI gene (Figure 1).

## Discussion:

The primary objective of this study was to find the allele and genotypic frequency of VDR BsmI gene polymorphism among CKD patients and controls. In this study, we demonstrate that VDR BsmI gene polymorphism is present among CKD patients in South Indian population. CKD patients with 'b' allele at intron 8 of chromosome 12q had higher serum creatinine and urea with decreased GFR than those with 'B' allele of BsmI gene. The data from meta-analysis study showed that VDR BsmI gene polymorphism was associated with chronic renal failure in Chinese and Spanish individuals [27]. Many studies showed a positive association between 'b' allele of BsmI gene polymorphism and development of hyperparathyroidism in ESRD patients [28, 29, 30, 31, 32]. The data from Italian study, found a highly significant association between BsmI polymorphism and development of left ventricular hypertrophy in ESRD patients [33]. The Indian study, found a strong association between BsmI polymorphism of VDR gene and CKD patients among North Indian population [1]. In contrast, the data from Egyptian study was unable to find any association between BsmI gene polymorphism and development of ESRD in patients on maintenance dialysis [34]. In our study, a statistically significant relationship was observed between b allele, Bb and bb genotypes of BsmI polymorphism and CKD patients in South Indian population. Limitations of the study are limited sample size, the study excluded CKD patients on peritoneal dialysis and there is no long-term follow-up of patients.

**Figure 3:**
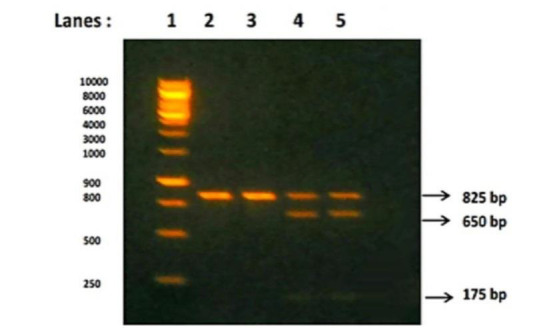
Agarose gel electrophoresis pattern of BsmI gene after restriction digestion in CKD patients

Agarose gel electrophoresis of cases and controls showing BsmI gene amplicon (825bp) before restriction digestion: lane 1 (1kb ladder), lanes 2 to 6 BsmI gene with 825bp Figure 3

Agarose gel electrophoresis of CKD patients showing PCR-restriction fragment length polymorphism analysis of BsmI gene after restriction digestion: Lanes 1 (1kb ladder), 2 and 3(BB) band size 825bp - Homozygous Wild genotype; lanes 4 and 5 (Bb) band sizes 825, 650 and 175bp - Heterozygous genotype; and lane 6 (bb) band sizes 650 and 175bp - Homozygous mutant genotype.

## Conclusion:

Our results suggest that BsmI polymorphism of VDR gene influence the risk of CKD development. BsmI polymorphism could be added to the list of potential markers to physicians in determining the CKD risk profile. Our findings suggest that genetic variation in BsmI gene has an impact on the development of CKD in the South Indian population. This study needs to be extended in a larger sample size to establish this genetic association more accurately.

## Figures and Tables

**Table 1:** Comparison of study parameters between cases and controls

	**Controls (n = 100)**	**Cases (n = 100)**	**p value**
**Study variables**			
Age (Years)	57.66 ±6.77	57.57 ±7.81	0.93
Gender (%)			
Male	53 (53%)	51 (51%)	
Female	47 (47%)	49 (49%)	
Blood Urea (mg/dl)	15.11 ±8.44	69.84 ±15.47	<0.0001*
Serum Creatinine (mg/dl)	0.59 ±0.26	3.93 ±1.66	<0.0001*
GFR(ml/min/1.73m2	90.89 ±20.22	35.78 ±25.45	<0.0001*
Data are represented in mean ± SD. n: Number of subjects, % : Percentage,GFR: Glomerular Filtration Rate.* considered as significant.

**Table 2:** Distribution of BsmI polymorphism among cases and controls

**Study participants**	**Genotypic frequency**			**Allele frequency**	
	**BB**	**Bb**	**bb**	**B**	b
Cases	7	23	70	0.19	0.81
(n = 100)	-7%	-23%	-70%	-19%	-81%

## References

[1] Tripathi G (2010). Ren Fail..

[2] Agarwal S (2009). Nephron Clin Pract..

[3] Kovesdy CP (2022). Kidney international supplements..

[4] Anupama YJ (2014). Indian J Nephrol..

[5] Singh AK (2013). BMC Nephrol..

[6] Kakitapalli YJ (2020). Kidney Dis (Basel)..

[7] Obrador GT (2011). Kidney Int Suppl.

[8] Kazancioglu R (2013). Kidney Int Suppl..

[9] Corredor Z (2020). Sci Rep..

[10]  Liwen C (2015). Biomedical reports.

[11] Cañadas-Garre M (2019). Front Genet..

[12] Owiredu William KBA (2020). Clinical hypertension.

[13] Kongsbak M (2013). Front Immunol..

[14] Ricca C (2018). Int J Mol Sci..

[15] Wan LY (2015). Molecules..

[16] Yamada S (2014). Trends Pharmacol Sci..

[17] Caccamo D (2020). PLoS One..

[18] Hussain T (2019). BMC Med Genet..

[19] Tian-Biao Zhou (2015). Journal of Receptors and Signal Transduction..

[20] Mallappallil M (2014). Clin Pract (Lond)..

[21] Becherucci F (2016). Clin Kidney J..

[22] Taneja N (2016). Asian Journal of Pharmaceutical and Clinical Research..

[23] Tobón-Arroyave SI (2017). J Clin Diagn Res..

[24] Goknar N (2016). Ren Fail..

[25] Gnanaprakash V (2019). J Diabetol..

[26] Fathy WM (2018). Menoufia Med J..

[27] Li L (2018). Ther Apher Dial..

[28] Pourfarzam M (2014). Adv Biomed Res..

[29] El-Shehaby AM (2013). Scand J Clin Lab Invest..

[30] Elshamaa MF (2022). Pediatr Endocrinol Diabetes Metab..

[31] Zhou TB (2015). J Recept Signal Transduct Res..

[32] Waziri B (2018). BMC Nephrol..

[33] Santoro D (2014). Nutrients..

[34] EL-Attar HA (2017). J Clini Nephrol..

